# The Impact of Thyme and Oregano Essential Oils Dietary Supplementation on Broiler Health, Growth Performance, and Prevalence of Growth-Related Breast Muscle Abnormalities

**DOI:** 10.3390/ani12213065

**Published:** 2022-11-07

**Authors:** Ahmed Zaazaa, Samer Mudalal, Ibrahim Alzuheir, Maen Samara, Nasr Jalboush, Adnan Fayyad, Massimiliano Petracci

**Affiliations:** 1 Department of Animal Production and Animal Health, Faculty of Agriculture and Veterinary Medicine, An-Najah National University, Nablus P.O. Box 7, Palestine; 2 Department of Nutrition and Food Technology, Faculty of Agriculture and Veterinary Medicine, An-Najah National University, Nablus P.O. Box 7, Palestine; 3 Department of Veterinary Medicine, Faculty of Agriculture and Veterinary Medicine, An-Najah National University, Nablus P.O. Box 7, Palestine; 4 Department of Agricultural and Food Sciences, Alma Mater Studiorum—University of Bologna, Piazza Goidanich 60, 47521 Cesena, Italy

**Keywords:** broiler performance, white striping, wooden, oregano oil, thyme oil

## Abstract

**Simple Summary:**

In recent years, there has been growing interest in the use of thyme and oregano essential oils in feed formulations to promote growth in chicken broilers. Thyme and oregano essential oils are considered promising ingredients to replace antibiotics as growth promotors. The aim of this study was to evaluate the impact of thyme and oregano essential oils on growth performance, broiler health, and the incidence of muscle abnormalities at different slaughter ages. This study showed that the addition of thyme and oregano essential oils, individually or in combination, significantly increased body weight compared to the control group. Thyme and oregano essential oils improved the feed conversion factor, which indicates lower feed intake (feed intake did not change according to our results) with higher meat production. Muscle abnormalities increased with the addition of thyme and oregano essential oils to broiler diets, which could be due to the increase in the growth rate. In conclusion, the inclusion of thyme and oregano oils in broiler chicken feed resulted in an improvement in the growth performance of broiler chickens.

**Abstract:**

The objective of this study was to investigate the effects of thyme and oregano essential oils (as growth promotors), individually and in combination, on the health, growth performance, and prevalence of muscle abnormalities in broiler chickens. Six hundred day-old Cobb 500 hybrid chickens were randomized into four dietary treatment groups with three replicates each. Chicks in the control group (C) received a basal diet, while the experimental treatment groups received basal diets containing 350 mg/kg of thyme oil (T1), 350 mg/kg of oregano oil (T2), and 350 mg/kg of thyme and oregano oil (T3). Growth performance parameters were evaluated at 14, 28, and 42 days. The broilers in treatments T1 and T2 had significantly higher body weights than the control group. The feed conversion ratio was the lowest in chicks who received oregano oil, followed by those fed thyme oil. The overall prevalence of growth-related breast muscle abnormalities (including white striping and white striping combined with wooden breast) in groups receiving essential oils (T1, T2, and T3) was significantly higher than in the control group (C). The thyme and oregano oil diets showed no significant differences in antibody titers against Newcastle disease or interferon-γ (INF-γ) serum levels. In conclusion, thyme and oregano oils had a positive impact on the growth performance of broiler chickens but increased the incidence of growth-related breast muscle abnormalities.

## 1. Introduction

Health concerns and regulatory restrictions on the use of antibiotics motivated the researchers to evaluate several alternatives to antibiotics. It was found that the use of different combinations of additives (such as medium-chain fatty acids, short-chain fatty acids, oregano essential oil, and sweet basil essential oil) exhibited positive effects on the growth performance of broilers [1]. Extracts of medicinal herbs (aromatic herbs) have received increasing attention from both researchers and producers as potential alternatives to conventional antibiotic growth promoters in broiler rations [2]. The beneficial effects of these essential oils as well as plant oils are related to their suitable chemical properties and functional groups, whose mechanisms of action remain to be explained [3,4]. Thyme and oregano essential oils have been extensively studied as feed supplements in broiler rations. However, varying results have been reported on their effects on overall broiler production performance [1,5]. There was no agreement between previous studies about the effects of thyme or oregano essential oils on feed intake, body weight gain, and feed conversion in broilers when these oils were used separately [6,7,8].

Extracts of thyme (*Thymus vulgaris*) and oregano (*Origanum vulgare* L.) are rich in several functional compounds such as carvacrol, thymol, lutein, and zeaxanthin, which play an important role in broiler health and growth performance [8,9]. The inclusion of oregano essential oil in broiler feed exhibited a protective effect against necrotic enteritis (NE) caused by *Clostridium perfringens* [9,10]. Some studies reported positive effects on the performance parameters of broiler chicks [8,11,12], while other studies showed no effect on broiler performance parameters [13,14]. In contrast to these studies, others reported negative effects of supplemental thyme or oregano oils in rations on broiler growth [6,7,15]. The use of thyme with prebiotics, such as mannan-oligosaccharides, in the feed formulation showed positive effects on the growth performance of broilers [16]. A few reports showed positive effects on the meat characteristics of carcasses when essential oils were added to the broiler rations [12,17]. These authors attributed the inconsistent results to differences in the doses of the essential oils used, environmental factors, the durations of the experiments, and health status of the chicks used.

Currently, poultry breeders and the meat industry are concerned about the occurrence of growth-related breast muscle abnormalities such as white striping (WS) and wooden breast (WB) [18]. In this context, several studies indicated that breast meat affected by these disorders had lower quality characteristics than normal breast meat [19,20,21,22,23]. Overall, the incidence rates of these abnormalities are alarming and appear to be unsustainable for the poultry industry [24]. It was found that the incidence of muscle abnormalities was higher in high-breast hybrids than standard-breast hybrids [25]. Moreover, the incidence of muscle abnormalities was higher in males than in females [26]. Incidence rates varied between studies. It was found that the incidence of WS was about 12% [25], while other researchers found that the incidence of WS reached 50% [27]. Another study showed that the incidence of WS was 75% in high-breast-yield hybrids and 74% in standard-breast-yield hybrids [28].

Mudalal et al. [29] examined the effect of a natural herbal extract on the occurrence of muscle abnormalities such as WS and WB. The results showed that the herbal extract reduced the occurrence of WS and WS combined with WB.

Most previous research results agreed that essential oils have antimicrobial [1,7,30], anticoccidial [13], antioxidant [31], antifungal [32], and chicken immune-boosting [13] effects.

In particular, Newcastle disease (ND) is considered one of the most serious diseases affecting broiler flocks worldwide, causing severe losses in the poultry sector [33]. Biosecurity and vaccination strategies are needed to control this disease [34]. Improving the immunization strategy of ND vaccines and host protection can be enhanced by complementary approaches, such as the use of herbal extracts from medicinal natural products [35]. There is growing evidence that the coadministration of herbal extracts with the vaccine showed increases in cytokine production and the antibody responses of immune cells [30].

To our knowledge, there are few studies that investigated the effects of thyme and oregano oils as a mixture on the health, growth performance, and prevalence of muscle abnormalities of broilers reared under commercial conditions. Therefore, the objective of this study was to examine the possible effects of thyme and oregano oils and a combination of both oils on the performance parameters, health status, and meat characteristics of broiler chicks as well as on the prevalence of muscle abnormalities from 1 day to 42 days of age.

## 2. Materials and Methods

### 2.1. Experimental Design

In this study, 600 one-day-old Cobb 500 hybrid broiler chicks were randomly divided into four groups of 150 chicks each, and each group was replicated three times. The chicks from the first treatment group received a basal ration (starter and grower) as a control group (C) (Table 1). The rations of the second treatment group (T1) were supplemented with 350 mg/kg of thyme essential oil. The rations of the third treatment group (T2) were supplemented with oregano essential oil at a concentration of 350 mg/kg. The rations of the fourth treatment group (T3) were supplemented with 350 mg/kg of thyme and oregano essential oils in equal proportions. In formulating each experimental ration, the essential oils were first mixed with the corresponding oil stock, and the mixture was then homogenized. The rations were mixed in two batches (the starters and the growers) and stored in airtight bags at room temperature for a short time before being fed to the chicks. The chicks were housed on a deep litter (fresh wood shavings) in an open-sided broiler house. Commercial protocols were used to rear the experimental chicks. The broiler house temperature was manipulated and closely monitored to avoid fluctuations, starting at 32 °C on day 1 and decreasing by 2 °C every week thereafter. The chicks were exposed to 24 h of lighting for the first 4 days and then 23 h of lighting and 1 h of darkness until the termination of the experiment. Chicks had access to feed and water around the clock. Body weight and feed intake were determined on days 14, 28, and 42. Mortality was recorded daily.

The feed conversion ratio was calculated as feed intake (g) per mean body weight (g) for each replicate of the treatment groups. The feed intake was calculated on a weekly basis, taking into account differences in feed weight. In addition, the weight of each broiler was recorded weekly.

The feed conversion ratio (FCR), body weight (BW), and feed intake (FI) were determined at different ages (days 14, 28, and 42).

### 2.2. Breast Weights

Seven broilers from each replicate were slaughtered at 42 days of age using a manual operation technique (*n* = 21/group). Breasts were weighed using a balance with a sensitivity of 0.01 g.

### 2.3. Assessment of Incidence of Growth-Related Breast Muscle Abnormalities

The incidence of growth-related breast muscle abnormalities was assessed at approximately 8 h postmortem. Muscle abnormalities were classified into three levels (normal, WS, and WB combined with WS) based on previously described criteria [27,36]. Breast fillets that exhibited no white striations or hardened areas were considered normal (N). Breast fillets that had white striations of varying thickness (thin to thick striations) were considered to be white-striped fillets (WS). Finally, breast fillets that had pale ridge-like bulges and diffuse hardened areas (namely WB) in combination with white striations were labeled as WS/WB.

The color trait (CIE L* = lightness, a* = redness, and b* = yellowness) of raw breast meat was measured in triplicate using a Chroma Meter CR-410 (Konica Minolta, Japan), and the skin-side surface of each fillet was considered a measuring point.

### 2.4. Newcastle Disease Vaccine Response

The freeze-dried live Newcastle Disease (ND) vaccine (LaSota strain-SPF origin vaccine, Biovac^®^, Cape Town, South Africa) was administrated via drinking water when the chicks were 12 days old, and this was repeated when the chicks were 22 days old. Blood samples were collected during the 1st, 3rd, and 5th weeks from the wing vein (*n* = 24). Each blood sample was left to coagulate at room temperature and was then centrifuged at 3000 rpm for 5 min.

### 2.5. Hemagglutination Inhibition (HI)

The collected sera were subjected to the hemagglutination inhibition (HI) test, and the level of the anti-NDV antibody titer was determined. The HI tests were performed in microplates using two-fold dilutions of serum, 1% PBS-washed chicken red blood cells, and four hemagglutinating units of vaccinal LaSota NDV (Biovac^®^, Cape Town, South Africa), following the method of Allan and Gough [37]. Titers were expressed as log2 values of the highest dilution that caused the inhibition of the hemagglutination. All tested serum samples were pretreated at 56 °C for 30 min to inactivate the nonspecific agglutinin.

### 2.6. ELISA Interferon Assay

The interferon concentration was determined by an immunoenzymatic assay (ELISA). At three time points (eight birds in each group at 7, 14, and 35 days) the serum level of interferon-γ (INF-γ) was determined using ELISA kits, following the instructions enclosed in the manufactured kits (Elabscience Co., Wuhan, China). Eight standards of 0, 15.6, 31.2, 62.5, 125, 250, 500, and 1000 pg/mL were added to the wells of the ELISA plate. Absorbance was measured at a wavelength of 450 nm. The interferon concentration was calculated using the standard curve.

### 2.7. Statistical Analysis

The effects of the thyme and oregano oils on the growth performance, feed conversion ratio, and the incidence of muscle abnormalities were assessed using an ANOVA (GLM procedure in SAS Statistical Analysis Software, version 9.1, 2002). Duncan’s test was employed to separate means in the case of the presence of statistical differences (*p* < 0.05). Pearson’s correlation was used to test the relationships between pairs of continuous variables (i.e., the feed conversion ratio, carcass, and visceral organ variables).

## 3. Results

The effects of thyme and oregano oils on the performance indices of broilers at different slaughter ages are shown in Table 2. Our results showed that the inclusion of thyme and/or oregano oils in feed did not exhibit any effect on feed intake. In general, there were significant differences in body weight between treatments at different slaughter ages (14, 28, and 42 days). Birds in treatment T2 (with oregano) exhibited the highest body weights and the lowest feed conversion ratios at different slaughter ages when compared to other groups. There were no significant differences between treatment C and T3 in these parameters. The birds in treatment T1 had higher body weights and lower feed conversion ratios than the birds of the control group (C) at different slaughter ages (14, 28, and 42 d).

**Figure 1:**
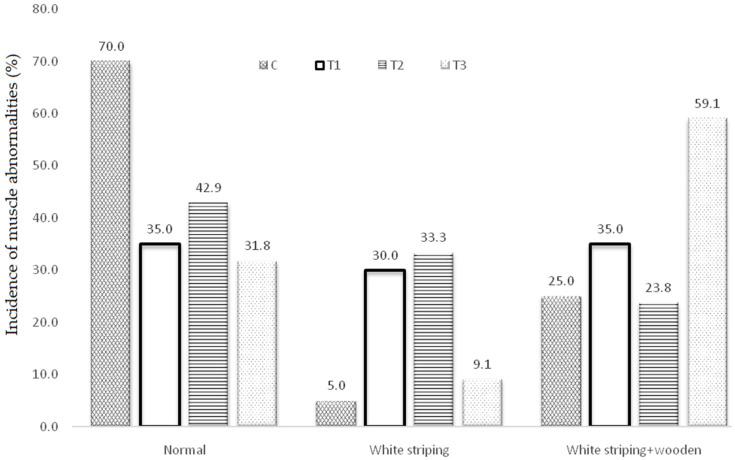
Percentages of normal, white striping, and white striping plus wooden breast meat abnormalities of broilers supplemented with herb extract (HE) (*n* = breasts/group). The basal diet (control, C) was similar to regular broiler starter diets, while the experimental treatments of the T1, T2, and T3 birds included the same diet as in the control group, but they were supplemented with herb extracts: thyme essential oil at 350 mg/kg (T1), oregano essential oil at 350 mg/kg (T2), and equal proportions of thyme and oregano essential oils at 350 mg/kg (T3).

The incidences of growth-related breast muscle abnormalities (normal, WS, and WS combined with WB condition) in all treatments are shown in Figure 1. The results showed that the control treatment had the highest percentage of normal cases (70%) compared with other treatments. Treatments T1 and T3 had quite similar percentages of normal cases, while treatment T2 had 42.9% normal cases, which was higher than treatment T1 and T2. The incidence of WS was the lowest (5%) in the control treatment compared to the other treatments. Treatment T2 exhibited the highest percentage of WS cases (33.3%), while treatments T1 and T3 had 30% and 9.1% FWS cases, respectively. For WS occurring with WB abnormalities, treatment T3 had the most cases (59.1%) compared with the other treatments. The control treatment and treatment T2 had quite similar percentages of the WB condition.

The effects of thyme and oregano extracts on color traits (L*, a*, and b*), pH, and breast weight are shown in Table 3. In general, there were no significant differences between treatments in the color index (L*, a*, and b*), pH, and breast weight. The effects of muscle abnormalities (normal, WS, and WS combined with WB) on the color traits (L*, a*, and b*), pH, and breast weight are shown in Table 4. Muscle abnormalities did not affect the color traits (L*, a*, and b*). Meat affected by the WB abnormality exhibited higher breast weight (213.22 vs. 188.97, *p* < 0.05) in comparison to normal meat, while white-striped meat exhibited intermediate values.

**Figure 2:**
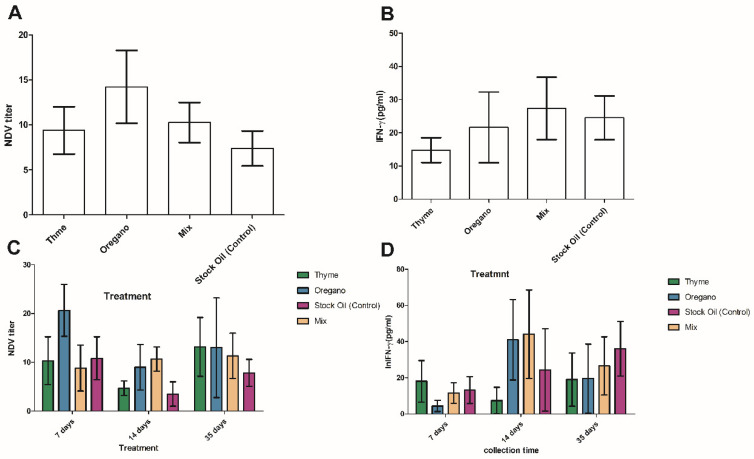
Cumulative changes in NDV Ab titer (**A**) and IFN-γ (**B**) and timely changes in NDV Ab titer (**C**) and IFN-γ (**D**) of the treated groups (at three time points: eight birds in each group at 7, 14, and 35 days). *p* > 0.05.

Dietary supplementation with thyme or oregano essential oils alone or a mixture had no significant (*p* < 0.05) positive effects on the broilers’ humoral or cellular immune reactions to NDV treatments (Figure 2). No significant effects were found for the treatments on the weekly and accumulative NDV Ab titers and IFN-γ levels of chicks during the experimental period (Figure 2).

## 4. Discussion

Thyme or oregano essential oils, when used as growth promoters, have been reported to improve body weight gain and feed conversion when added to broiler rations [7,8,17]. In the present study, essential oils of thyme or oregano at a dosage of 350 mg/kg significantly increased the average body weight at 14, 28, and 42 days of age. A similar trend was observed in the feed conversion ratio. The results of the present study were in disagreement with the results of some previous studies that revealed that thyme or oregano oils did not affect body weight gain and feed efficiency [8,11,17]. It has also been suggested that dietary supplementation with oregano or thyme oils may exert positive effects on growth parameters when relatively high doses are used [38]. However, other studies concluded that incremental doses of 100 to 1000 mg/kg or 300 to 1200 mg/kg of oregano oils did not always improve production performance [6,15,39]. These contrasting observations could be explained by differences in the concentrations and chemical compositions of the oils used, the lengths of the experimental periods, the numbers of chicks used, and management factors. In the present study, the variation in these factors was minimized to some extent so that the differences in the performance parameters could only be attributed to the supplemental oils.

Saleh et al. [39] reported that the feed intake of chicks that received thyme essential oil (100 to 200 mg/kg) was higher than that of chicks in a control treatment. These findings were in disagreement with the results of the present study. In contrast, Wade et al. [8] reported that supplementing broiler diets with varying amounts of thyme oil had no effect on feed intake.

Regarding the effect of herbal extract addition on the incidence of growth-related breast muscle abnormalities, our results were partially in agreement with previous studies. Mudalal et al. [29] found that the incidence of WS was 19.5–39.2% and that WS combined with WB was in the range of 67–76.5% at a slaughtering age of 41 days. Previous studies showed that the incidence of WS was 25.7–32.3% [20]. Cruz et al. [40] found that the prevalence of WS and WB abnormalities ranged from 32.3 to 89.2%. Mudalal [41] found that the total prevalence of WS in turkey breast was 61.3%. Mudalal and Zaazaa [23] showed that the incidence of muscle abnormalities was highly affected by slaughter age, where it was about 45% at a slaughter age of 34 days and 100% at a slaughter age of 48 days.

The overall results showed that the addition of thyme and oregano extracts to broiler diets increased the incidence of these abnormalities. The overall prevalence of muscle abnormalities (WS and WS combined with WB) was higher in the treated groups (T1, T2, and T3) than in the control group (65%, 57.1%, 68.2% vs. 30%), respectively. These results may be attributed to an increase in the growth rate and the live weight of broilers at slaughter (Table 2). Previous studies have shown that an increase in growth rate was associated with a higher prevalence of muscle abnormalities [19,28,42,43].

The addition of thyme and oregano extracts exhibited no effects on the color traits (L*, a*, and b*), pH, and breast weight. The incidence of muscle abnormalities (normal, WS, and WS combined with WB) had no effect on the color traits (L*, a*, and b*) and pH but affected breast weight. Zambonelli et al. [44] found that WS combined with WB did not affect the a* and b* values, while the L* values were lower than in normal meat. Another study found that meat with WS alone or combined with WB abnormalities did not affect the color traits (L*, a*, and b*) [45]. Even though there was an apparent increase in pH due to the presence of muscle abnormalities, it was not significant. In this context, Tijare et al. [20] found that the WS abnormality did not affect pH values, while Soglia et al. [19] showed that meat affected by both abnormalities (WS and WB) exhibited a higher pH than normal meat.

Meat affected by the WB abnormality exhibited a higher breast weight (213.2 vs. 189.0 g, *p* < 0.05) compared to normal meat, while white-striped meat exhibited intermediate values. Similar results were obtained by Tasoniero et al. [46], where WB exhibited significantly higher breast weight than normal meat while white-striped meat exhibited moderate values. In addition, Malila et al. [47] found that meat affected by the WB abnormality had a higher breast weight than normal meat.

Dietary supplementation with thyme or oregano essential oils alone or in a mixture had no significant (*p* < 0.05) positive effects on the humoral or cellular immune reactions of broilers to NDV in the treated groups (Figure 2). No significant effects of the treatments were detected in the weekly and cumulative NDV-Ab titers and IFN-γ levels of the chicks during the experimental period. Our results were also in agreement with previous studies [30,48] that used thyme in the feed and drinking water of broilers and found no significant differences in antibody titers against NDV compared to the control group. In contrast, our results contradict previous reports in which thyme essential oil supplementation (135 mg/kg of feed) increased the humoral immune response against NDV compared to the control group [39]. Since thyme has been reported to have antibacterial and antifungal activities and the main components of thyme are thymol and carvacrol, which are reported to have strong antioxidant properties, an increase in the immune responses of the chicks was expected [48,49]. Although the dietary treatments had no significant effects on the immune-related parameters measured in this study, no deleterious effects were observed from the addition of thyme, oregano, or a combination to the diet. This could be due to the quantity of the additives used in our study. The results also showed that broilers whose diets were supplemented with thyme and oregano or a mixture of both showed no change in the production of IFN-γ proinflammatory cytokines compared with the control group. No significant differences were observed in the relative expression levels of IFN-γ. This is consistent with results published by Hassan and Awad [50], who claimed that thyme supplementation did not alter relative messenger RNA (mRNA) transcription levels for IFN-γ and other cytokines. Moreover, thymol inhibited the phosphorylation of NFκB and decreased the production of IL-6, TNF-α, iNOS, and COX-2 in LPS-stimulated mouse epithelial cells [51]. These findings support the previously mentioned results and indicate that the anti-inflammatory effects of thyme and oregano make them suitable for use in animal production. On the other hand, it was found that oregano oil combined with a macleaya cordata oral solution improved serum immunological characteristics [52].

## 5. Conclusions

In conclusion, the addition of oregano oil was the most effective in improving the growth performance of broiler chickens and was better than thyme oils. The inclusion of thyme and oregano essential oils together had no positive impact on broiler health. While the essential oils of oregano and thyme improved the feed conversion factor, the incidence of muscle abnormalities increased, and this may be attributed to the increase in the growth rate. Therefore, it is important to consider the impact of these muscle abnormalities on meat quality when developing any growth promotion program.

## Figures and Tables

**Table 1:** Composition of the basal diets fed to broilers in the feeding trial, g/kg.

Ingredient (g/kg)	Starter	Grower
Yellow corn	316	351
Soybean meal	300	249
Wheat	250	250
Sunflower	50	60
Oil	41	52
DCP ^1^	16.5	14.5
Limestone	13.0	11.5
NaCl	2.5	2.5
Premix ^2^	4	4
DL-methionine	2.5	1.9
L-lysine	4.5	4.0
Threonine	1	0.4
Sodium bicarbonate	1	1
Calculated analysis (%)		
Crude protein	22.0	20.0
Lysine	1.10	1.10
Methionine	0.55	0.56
Calcium	1.00	1.10
Available P	46	47
ME, Kcal/kg	3100	3000

^1^ Dicalcium phosphate. ^2^ Premix/kg diet: vitamin A, 12,000 IU; vitamin D3, 1500 IU; vitamin E, 50 mg; vitamin K3, 5 mg; vitamin B1, 3 mg; vitamin B2, 6 mg; vitamin B6, 5 mg; vitamin B12, 0.03 mg; niacin, 25 mg; Ca-D-pantothenate, 12 mg; folic acid, 1 mg; D-biotin, 0.05 mg; apo-carotenoic acid ester, 2.5 mg; choline chloride, 400 mg; manganese, 100 g; zinc, 100 g; iron, 40 g; copper, 15 g; iodine, 1 g; cobalt, 0.2 g; selenium, 0.35 g; wheat enzyme, 100 g; phytase, 750 kfu; lasalocid, 100 g; BMD, 55 g.

**Table 2:** Effects of oils on performance indices of broilers at different ages.

		C M ± STD	T1 M ± STD	T2 M ± STD	T3 M ± STD	*p* Value
Cumulative feed intake (g/bird)	14 d	491.00 ± 0.00	493.67 ± 0.01	496.67 ± 0.01	493.33 ± 0.00	0.50
	28 d	1838.00 ± 0.03	1820.67 ± 0.01	1827.33 ± 0.01	1821.33 ± 0.01	0.57
	42 d	4014.00 ± 0.01	3995.67 ± 0.02	4004.67 ± 0.02	4016.00 ± 0.03	0.53
Bodyweight (g)	14 d	400.71 ^c^ ± 0.01	413.42 ^b^ ± 0.01	425.99 ^a^ ± 0.00	400.99 ^c^ ± 0.00	<0.05
	28 d	1148.72 ^c^ ± 0.01	1161.92 ^b^ ± 0.00	1176.38 ^a^ ± 0.00	1147.98 ^c^ ± 0.011	<0.05
	42 d	1965.58 ^c^ ± 0.01	2009.90 ^b^ ± 0.01	2031.97 ^a^ ± 0.00	1971.14 ^c^ ± 0.01	<0.05
Feed conversion ratio	14 d	1.23 ^a^ ± 0.02	1.20 ^b^ ± 0.01	1.17 ^c^ ± 0.00	1.23 ^a^ ± 0.01	<0.05
	28 d	1.60 ^a^ ± 0.02	1.57 ^bc^ ± 0.01	1.55 ^c^ ± 0.00	1.59 ^ab^ ± 0.01	<0.05
	42 d	2.04 ^a^ ± 0.01	1.99 ^b^ ± 0.01	1.97 ^c^ ± 0.00	2.04 ^a^ ± 0.01	<0.05

Data are reported as means (M, *n* = 150/group) and standard deviations (STD). Different letters in the same row indicate significant differences (*p* < 0.05). Treatment C: basal ration as a control group, treatment T1: basal ration supplemented with 350 mg/kg of thyme essential oil, treatment T2: basal ration supplemented with 350 mg/kg of oregano essential oil, treatment T3: basal ration supplemented with 350 mg/kg of thyme and oregano essential oils in equal proportions.

**Table 3:** The effects of the inclusion of thyme and oregano extracts on color traits (L*, a*, and b*), pH, and breast weight for raw chicken breast.

	C M ± STD	T1 M ± STD	T2 M ± STD	T3 M ± STD	*p* Value
L* value *	70.72 ± 3.59	68.07 ± 5.52	68.33 ± 5.09	68.78 ± 8.09	0.44
a* value *	2.24 ± 1.31	2.19 ± 1.70	2.68 ± 1.36	2.01 ± 0.73	0.42
b* value *	4.29 ± 1.74	4.22 ± 1.62	4.10 ± 1.51	4.65 ± 1.91	0.40
pH	5.92 ± 0.24	5.92 ± 0.08	5.87 ± 0.09	5.88 ± 0.09	0.57
Breast weight (g)	197.8 ± 42.4	191.6 ± 43.4	212.9 ± 31.0	202.3 ± 26.3	0.29

Data are reported as means (M, *n* = 21/group) and standard deviations (STD). Treatment C: basal ration as a control group, treatment T1: basal ration supplemented with 350 mg/kg of thyme essential oil, treatment T2: basal ration supplemented with 350 mg/kg of oregano essential oil, treatment T3: basal ration supplemented with 350 mg/kg of thyme and oregano essential oils in equal proportions. * The color trait (CIE L* = lightness, a* = redness, and b* = yellowness).

**Table 4:** The effects of muscle abnormalities (normal, white striping (WS), and white striping combined with wooden breast condition (WS and WB)) on the color traits (L*, a*, and b*), pH, and breast weight.

	Normal M ± STD	WS M ± STD	WS and WB M ± STD	*p* Value
L* value *	70.15 ± 4.45	69.22 ± 8.63	67.60 ± 5.55	0.20
a* value *	2.51 ± 1.45	1.83 ± 1.28	2.02 ± 0.79	0.11
b* value *	4.25 ± 1.62	3.73 ± 1.19	4.52 ± 1.64	0.31
pH	5.87 ± 0.18	5.94 ± 0.08	5.91 ± 0.08	0.22
Breast weight (g)	189.0 ± 38.8 ^a^	205.9 ± 30.0 ^ab^	213.2 ± 30.9 ^b^	<0.05

Data are reported as means (M, *n* = 21/group) and standard deviations (STD). Different letters in the same row indicate significant differences (*p* < 0.05). The levels of white striping (WS) were classified as normal, moderate, or severe according to Kuttappan et al. [27]. * The color trait (CIE L* = lightness, a* = redness, and b* = yellowness).

## Data Availability

The data presented in this study are available on request from the corresponding author. The data are not publicly available.

## References

[1] Supchukun K., Yata T., Israsena Na Ayudhya P., Angkanaporn K. (2022). Key Success Factors for the Development of Innovative Antibiotic Replacement Products to Accelerate Growth in Broilers. Sustainability.

[2] Puvača N., Tufarelli V., Giannenas I. (2022). Essential Oils in Broiler Chicken Production, Immunity and Meat Quality: Review of *Thymus vulgaris*, *Origanum vulgare*, and *Rosmarinus officinalis*. Agriculture.

[3] Nazzaro F., Fratianni F., De Martino L., Coppola R., De Feo V. (2013). Effect of essential oils on pathogenic bacteria. Pharmaceuticals.

[4] Abo Omar J., Zaazaa A. (2016). Performance and Carcass Characteristics of Rabbits Fed Oil Supplemented Diets. J. Sci. Technol..

[5] Alagawany M., Abd El-Hack M., Farag M., Shaheen H., Abdel-Latif M., Noreldin A., Patra A. (2018). The usefulness of oregano and its derivatives in poultry nutrition. Worlds Poult Sci. J..

[6] Demir E., Kilinc K., Yildirim Y., Dincer F., Eseceli H. (2008). Comparative effects of mint, sage, thyme and flavomycin in wheat-based broiler diets. Arch. Zootech..

[7] Vlaicu P.A., Untea A.E., Turcu R.P., Saracila M., Panaite T.D., Cornescu G.M. (2022). Nutritional Composition and Bioactive Compounds of Basil, Thyme and Sage Plant Additives and Their Functionality on Broiler Thigh Meat Quality. Foods.

[8] Wade M., Manwar S., Kuralkar S., Waghmare S., Ingle V., Hajare S. (2018). Effect of thyme essential oil on performance of broiler chicken. J. Entomol. Zool. Stud..

[9] Jin X., Huang G., Luo Z., Hu Y., Liu D. (2022). Oregano (*Origanum vulgare* L.) Essential Oil Feed Supplement Protected Broilers Chickens against *Clostridium perfringens* Induced Necrotic Enteritis. Agriculture.

[10] Attia Y., Bakhashwain A., Bertu N. (2018). Utilisation of thyme powder (*Thyme vulgaris* L.) as a growth promoter alternative to antibiotics for broiler chickens raised in a hot climate. Eur. Poult. Sci..

[11] Mathlouthi N., Bouzaienne T., Oueslati I., Recoquillay F., Hamdi M., Urdaci M., Bergaoui R. (2012). Use of rosemary, oregano, and a commercial blend of essential oils in broiler chickens: In vitro antimicrobial activities and effects on growth performance. J. Anim. Sci..

[12] Peng Q., Li J., Li Z., Duan Z., Wu Y. (2016). Effects of dietary supplementation with oregano essential oil on growth performance, carcass traits and jejunal morphology in broiler chickens. Anim. Feed Sci. Technol..

[13] Alp M., Midilli M., Kocabağlı N., Yılmaz H., Turan N., Gargılı A., Acar N. (2012). The effects of dietary oregano essential oil on live performance, carcass yield, serum immunoglobulin G level, and oocyst count in broilers. J. Appl. Poult. Res..

[14] Koiyama N.T.G., Rosa A.P., Padilha M.T.S., Boemo L.S., Scher A., Melo Amd S., Fernandes M.O. (2014). Desempenho e rendimento de carcaça de frangos de corte alimentados com mistura de aditivos fitogênicos na dieta. Pesqui. Agropecu. Bras..

[15] Zeng Z., Zhangm S., Wang H., Piao X. (2015). Essential oil and aromatic plants as feed additives in non-ruminant nutrition: A review. J. Anim. Sci. Biotechnol..

[16] Amouei H., Ferronato G., Qotbi A.A.A., Bouyeh M., Dunne P.G., Prandini A., Seidavi A. (2021). Effect of Essential Oil of Thyme (*Thymus vulgaris* L.) or Increasing Levels of a Commercial Prebiotic (TechnoMOS^®^) on Growth Performance and Carcass Characteristics of Male Broilers. Animals.

[17] Eler G., Gomes A., Trindade B., Almeida L., Dilelis F., Cardoso V., Lima C. (2019). Oregano essential oil in the diet of broilers: Performance, carcass characteristics, and blood parameters. S. Afr. J. Anim. Sci..

[18] Petracci M., Soglia F., Berri C., Petracci M., Berri C. (2017). Muscle Metabolism and Meat Quality Abnormalities, in Poultry Quality Evaluation.

[19] Soglia F., Laghi L., Canonico L., Cavani C., Petracci M. (2016). Functional property issues in broiler breast meat related to emerging muscle abnormalities. Food Res. Int..

[20] Tijare V.V., Yang F.L., Kuttappan V.A., Alvarado C.Z., Coon C.N., Owens C.M. (2016). Meat quality of broiler breast fillets with white striping and woody breast muscle myopathies. Poult. Sci..

[21] Mudalal S., Zaid A., Abu-Khalaf N., Petracci M. (2020). Predicting the quality traits of white striped turkey breast by visible/near infra-red spectroscopy and multivariate data analysis. Ital. J. Anim. Sci..

[22] Zaid A., Abu-Khalaf N., Mudalal S., Petracci M. (2020). Differentiation between Normal and White Striped Turkey Breasts by Visible/Near Infrared Spectroscopy and Multivariate Data Analysis. Food Sci. Anim. Resour..

[23] Mudalal S., Zaazaa A. (2022). Influence of Slaughter Age on the Occurrence and Quality Characteristics of White Striping and Wooden Muscle Abnormalities. Food Sci. Anim. Resour..

[24] Soglia F., Mazzoni M., Petracci M. (2019). Spotlight on avian pathology: Current growth-related breast meat abnormalities in broilers. Avian Pathol..

[25] Petracci M., Mudalal S., Bonfiglio A., Cavani C. (2013). Occurrence of white striping under commercial conditions and its impact on breast meat quality in broiler chickens. Poult. Sci..

[26] Kuttappan V.A., Huff G.R., Huff W.E., Hargis B.M., Apple J.K., Coon C., Owens C.M. (2013). Comparison of hematologic and serologic profiles of broiler birds with normal and severe degrees of white striping in breast fillets. Poult. Sci..

[27] Kuttappan V.A., Brewer V.B., Apple J.K., Waldroup P.W., Owens C.M. (2012). Influence of growth rate on the occurrence of white striping in broiler breast fillets. Poult. Sci..

[28] Trocino A., Piccirillo A., Birolo M., Radaelli G., Bertotto D., Filiou E., Petracci M., Xiccato G. (2015). Effect of genotype, gender and feed restriction on growth, meat quality and the occurrence of white striping and wooden breast in broiler chickens. Poult. Sci..

[29] Mudalal S., Zaazaa A., Omar J.A. (2021). Effects of Medicinal Plants Extract with Antibiotic Free Diets on Broilers Growth Performance and Incidence of Muscles Abnormalities. Braz. J. Poult. Sci..

[30] Rahimi S., Teymori Zadeh Z., Torshizi K., Omidbaigi R., Rokni H. (2011). Effect of the three herbal extracts on growth performance, immune system, blood factors and intestinal selected bacterial population in broiler chickens. J. Agric. Sci. Technol..

[31] Biswas A., Chatli M., Jairath G. (2017). Natural Antioxidants. Natural Antioxidants in Poultry Products.

[32] Bedoya-Serna C.M., Dacanal G.C., Fernandes A.M., Pinho S.C. (2018). Antifungal activity of nanoemulsions encapsulating oregano (*Origanum vulgare*) essential oil: In vitro study and application in Minas Padrao cheese. Braz. J. Microbiol..

[33] Alexander D.J. (2000). Newcastle disease and other avian paramyxoviruses. Rev. Sci. Tech..

[34] Sharif A., Ahmad T., Umer M., Rehman A., Hussain Z. (2014). Prevention and control of Newcastle disease. Int. J. Agric. Innov. Res..

[35] Helal M.S., Youssef F.M., Moursi M.K., Khalil W.F., Abdel-Daim M.M. (2015). Effectiveness of prebiotic as an alternative to the antimicrobial growth promoter on growth performance, blood constituents, intestinal healthiness and immunity of broilers. Alex. J. Vet. Sci..

[36] Sihvo H.K., Immonen K., Puolanne E. (2014). Myodegeneration with fibrosis and regeneration in the pectoralis major muscle of broilers. Vet. Pathol..

[37] Allan W., Gough R.A. (1974). Standard haemagglutination inhibition test for Newcastle disease. A comparison of macro and micro methods. Vet. Rec..

[38] El-Ghousein S.S., Al-Beitawi N.A. (2009). The effect of feeding of crushed thyme (*Thymus valgaris* L) on growth, blood constituents, gastrointestinal tract and carcass characteristics of broiler chickens. Poult. Sci. J..

[39] Saleh N., Allam T., El-Latif A., Ghazy E. (2014). The effects of dietary supplementation of different levels of thyme (*Thymus vulgaris*) and ginger (*Zingiber officinale*) essential oils on performance, hematological, biochemical and immunological parameters of broiler chickens. Glob. Vet..

[40] Cruz R.F., Vieira S.L., Kindlein L., Kipper M., Cemin H.S., Rauber S.M. (2017). Occurrence of white striping and wooden breast in broilers fed grower and finisher diets with increasing lysine levels. Poult. Sci..

[41] Mudalal S. (2019). Incidence of White Striping and Its Effect on the Quality Traits of Raw and Processed Turkey Breast Meat. Food Sci. Anim. Resour..

[42] Griffin J.R., Moraes L., Wick M., Lilburn M.S. (2018). Onset of white striping and progression into wooden breast as defined by myopathic changes underlying Pectoralis major growth. Estimation of growth parameters as predictors for stage of myopathy progression. Avian. Pathol..

[43] Sihvo H.K., Airas N., Linden J., Puolanne E. (2018). Pectoral Vessel Density and Early Ultrastructural Changes in Broiler Chicken Wooden Breast Myopathy. J. Comp. Pathol..

[44] Zambonelli P., Zappaterra M., Soglia F., Petracci M., Sirri F., Cavani C., Davoli R. (2016). Detection of differentially expressed genes in broiler pectoralis major muscle affected by White Striping–Wooden Breast myopathies. Poult. Sci..

[45] Mudalal S., Lorenzi M., Soglia F., Cavani C., Petracci M. (2015). Implications of white striping and wooden breast abnormalities on quality traits of raw and marinated chicken meat. Animal.

[46] Tasoniero G., Cullere M., Cecchinato M., Puolanne E., Dalle Zotte A. (2016). Technological quality, mineral profile, and sensory attributes of broiler chicken breasts affected by White Striping and Wooden Breast myopathies. Poult. Sci..

[47] Malila Y., U-Chupaj J., Srimarut Y., Chaiwiwattrakul P., Uengwetwanit T., Arayamethakorn S., Punyapornwithaya V., Sansamur C., Kirschke C.P., Huang L. (2018). Monitoring of white striping and wooden breast cases and impacts on quality of breast meat collected from commercial broilers (*Gallus gallus*). Asian Australas J. Anim. Sci..

[48] Toghyani M., Tohidi M., Gheisari A.A., Tabeidian S.A. (2010). Performance, immunity, serum biochemical and hematological parameters in broiler chicks fed dietary thyme as alternative for an antibiotic growth promoter. Afr. J. Biotechnol..

[49] Kheiri F., Faghani M., Landy N. (2018). Evaluation of thyme and ajwain as antibiotic growth promoter substitutions on growth performance, carcass characteristics and serum biochemistry in Japanese quails (*Coturnix japonica*). Anim. Nutr..

[50] Hassan F.A., Awad A. (2017). Impact of thyme powder (*Thymus vulgaris* L.) supplementation on gene expression profiles of cytokines and economic efficiency of broiler diets. Environ. Sci. Pollut. Res..

[51] Liang D., Li F., Fu Y., Cao Y., Song X., Wang T., Wang W., Guo M., Zhou E., Li D. (2014). Thymol inhibits LPS-stimulated inflammatory response via down-regulation of NF-κB and MAPK signaling pathways in mouse mammary epithelial cells. Inflammation.

[52] Zhang C., Li W., Chen L., Chen Z., Wang X., Xu Q., Zhang H., Chen H., Liu J. (2022). Oregano Oil Combined with Macleaya Cordata Oral Solution Improves the Growth Performance and Immune Response of Broilers. Animals.

